# Mental health problems in male young offenders in custodial versus community based-programs: implications for juvenile justice interventions

**DOI:** 10.1186/s13034-016-0131-6

**Published:** 2016-11-01

**Authors:** Daniel Rijo, Nélio Brazão, Ricardo Barroso, Diana Ribeiro da Silva, Paula Vagos, Ana Vieira, Ana Lavado, Ana Margarida Macedo

**Affiliations:** 1 Research Unit of the Cognitive-Behavioral Research and Intervention Center, Faculty of Psychology and Education Sciences, University of Coimbra, Rua do Colégio Novo, 3001-802 Coimbra, Portugal; 2 University of Trás-os-Montes and Alto Douro, Vila Real, Portugal; 3 Center for Psychology at University of Porto, Porto, Portugal; 4 General Directorship of Social Reinsertion and Prison Services of the Portuguese Ministry of Justice, Lisbon, Portugal

**Keywords:** Prevalence rates, Mental health problems, Male young offenders, Custodial versus community-based programs, Juvenile justice interventions

## Abstract

**Background:**

Young offenders are known to be a population with high prevalence of mental health disorders. In most cases, these disorders are neither identified nor treated properly, with the majority of them being chronic and difficult to treat. In many countries, the prevalence rates of psychopathology in male young offenders are still unknown and no psychotherapeutic interventions are delivered. Therefore, the main goal of the present study was to assess mental health problems in Portuguese male young offenders placed in either custodial or community-based programs and discuss treatment implications within the juvenile justice interventions.

**Methods:**

Participants in this study included 217 male young offenders aged between 14 and 20 years old that were randomly selected using a random number table. From the total sample, 122 (56.3 %) participants were placed in juvenile detention facilities, and 95 (43.7 %) were receiving community-based programs. Participants were interviewed with the Mini-International Neuropsychiatric Interview for Children and Adolescents, a structured interview that assesses DSM-IV Axis I Mental Disorders. Participants aged 18 years or older were also assessed with the antisocial personality disorder section from the Structured Clinical Interview for DSM-IV Axis II Personality Disorders.

**Results:**

Results showed a high prevalence of mental health disorders, with a global prevalence of 91.2 % in the total sample. In both groups, global prevalence rates were equally high (93.4 % in youth in custodial versus 88.4 % in youth in community-based programs). Substance-related disorders were more prevalent in youth placed in juvenile facilities, whereas anxiety and mood disorders were more often found in the community-based group. Moreover, oppositional defiant disorder was more prevalent in youth from the community, whereas antisocial personality disorder and conduct disorder were less prevalent than expected in this same group. A high comorbidity rate was also found, with the majority of participants from both groups’ fulfilling criteria for two or more disorders. Additionally, participants with conduct disorder were over four times more likely to fulfill criteria for substance abuse.

**Conclusions:**

Our findings inform about specific needs concerning mental health intervention that should be taken into account when deciding and planning rehabilitation programs for male young offenders, either from custodial or community-based programs.

## Background

Research on juvenile offenders has consistently identified an overlap between criminal behavior and mental health problems, and has begun to clarify the links between antisocial behavior and psychopathology [1]. A considerable amount of research has studied the prevalence rates of psychopathology in male youth intervened by the juvenile justice systems in different countries. Results have shown that male young offenders tend to present substantially higher rates of both externalized and internalized disorders, when compared with normative peers [2–5].

Although a considerable variability in the prevalence of mental health disorders is found across studies, research stresses out that 60–95 % of male young offenders meet criteria for, at least, one psychiatric disorder [1, 3, 4, 6]. As expected, disruptive disorders were the most frequently reported diagnoses in juvenile justice samples, with conduct disorder being the most frequent diagnosis among male young offenders, with prevalence rates ranging from 31 to 100 % [5, 7]. Antisocial personality disorder is also frequently found in male young offenders, with prevalence rates ranging from 76 to 81 % [4, 6]. A recent study [8] further shown that male young offenders with personality disorders have high levels of anger–irritability, aggression, delinquency, distress, and reduced restraint, when compared with young offenders without personality pathology. Other than conduct disorder and/or antisocial personality disorder, male young offenders still present considerably high rates of psychopathology. Another diagnosis frequently related with antisocial behavior is attention-deficit hyperactivity disorder [9]. A recent meta-analysis reported that there is a fivefold increase in the prevalence of attention-deficit hyperactivity disorder in male detained youth (30.1 %), when compared with peers from the community [10].

It is well established that physical, emotional, and/or sexual trauma exposure is highly prevalent among male juvenile offenders [4, 11–14]. Abram and colleagues [11] found that 92.5 % of young offenders had been exposed to, at least, one type of trauma, and most of them experienced several traumatic events. Nevertheless, the authors found that only 11.2 % of young offenders met criteria for post-traumatic stress disorder.

Substance-related disorders are also reported as common among male young offenders, with prevalence rates ranging between 30 and 56 % [7, 15–17]. The relationship between mood disorders, namely depression, and antisocial behavior has also been studied, and longitudinal research suggests that depressive symptoms during adolescence might predict later antisocial behavior [18]. It is worth noting that anxiety disorders showed to have a prevalence rate of about 30 % [17] in male young offenders.

Prevalence studies have also stressed out that psychiatric comorbidity is the norm among male young offenders; 46–80 % of these individuals meet criteria for more than one psychiatric disorder [1, 4, 5, 7, 15, 19]. Particularly, the presence of a substance-related disorder seems to increase the already high likelihood of having a comorbid disorder [15]. Teplin and colleagues [20] found that 20 % of male young offenders diagnosed with a substance-related disorder had a comorbid mental disorder, most commonly attention-deficit hyperactivity disorder, but also frequently an anxiety or a mood disorder. A longitudinal large-scale study found a high comorbidity and continuity of psychiatric disorders among male youth 5 years after detention, especially for those with multiple disorders at baseline [15]. The authors highlighted that, although the comorbidity rates seemed to decrease in youth after detention, they remain significantly higher than those found in the general population.

It should also be noticed that psychopathology is considered a risk factor for recidivism both in adult inmates [21, 22] as well as in juvenile offenders [23, 24]. Concerning youth, disruptive disorders and/or substance-related disorders (isolated or in comorbidity with other mental health problems) seem to play a major predictive role in reoffending [23]. A longitudinal study found that substance-related disorders were the strongest predictors of subsequent violence in male young offenders after detention [25].

Despite these findings, some authors found that a great proportion of young offenders do not receive appropriate treatment [17]. In a recent study, Burke et al. [26] found that relatively few youth (approximately 20 %) were in contact with mental health services. This is especially relevant, since it is well established that antisocial individuals tend to have a better response to treatment in early developmental stages, such as adolescence [27, 28].

Studies on the prevalence of mental health problems among young offenders were mainly conducted in the United States of America, remaining scarce in European countries. Moreover, previous studies present several methodological flaws, namely: (a) the use of small or unrepresentative samples, which provides less reliable prevalence rates [1]; (b) the lack of randomized samples, with most studies using convenience samples or samples of youth already referred as having mental health problems [29]; (c) measurement inconsistency, with studies using semi-structured interviews [3], self-report questionnaires [30], or data from courts or psychiatric records [31]; (d) measurement reliability, with some studies using well-standardized instruments, such as structured clinical interviews, but others relying on unstandardized measurement tools with less empirical validation [1]; and, finally, (e) very few studies are focused on comparing psychopathology prevalence rates in young offenders in custodial versus community-based programs [32].

The current study tried to overcome some of these methodological flaws. It is also the first study on mental health problems with Portuguese male young offenders, thus adding to research on this issue in European countries. The main goals of this study were, firstly, to assess the prevalence rates of mental health disorders in a randomized sample of male young offenders intervened by the Portuguese Juvenile Justice System, using structured clinical interviews. Secondly, the prevalence rates of mental health disorders were compared in two different groups: youngsters placed in juvenile facilities versus youngsters placed in community-based programs.

## Methods

### Participants

Participants in this study were male young offenders, aged between 14 and 20 years old. Participants were recruited from a wider research project aiming to study the prevalence rates of mental health disorders among youth intervened by the Portuguese Juvenile Justice, and to propose specific psychotherapeutic interventions to address the mental health problems of male young offenders. Participants with cognitive impairment (according to data collected from the justice report files), psychotic symptoms and/or developmental disorders (both assessed with the clinical interview for Axis I disorders used in this research; for a description of the interview, see the “Measures” section), were not included in this study. These exclusion criteria were applied because subjects with this kind of diagnosis require particular interventions already provided by specific mental health professionals and institutions collaborating with the Portuguese Juvenile Justice System. Female young offenders were also excluded because they represent only 10–15 % of the young offenders intervened by the Portuguese Juvenile Justice System, and any possible idiosyncrasies from this cohort would be underrepresented.

According to the Portuguese Ministry of Justice [33], there was a total of 2545 youth intervened by the Portuguese Justice System at the time of data collection, being 2193 male. Of those 2193 male young offenders, 591 were placed in community-based programs and 235 were placed in juvenile detention facilities [33]. It is important to highlight that, according to the Portuguese legal system, these are the two more severe consequences a court can apply to youth aged between 12 and 16 years’ old who have committed an offense. In general, severe offenses (e.g., aggravated assault, sexual assault, kidnapping, attempted homicide, homicide) lead the court to decide for youth to be placed in a juvenile detention facility rather than in a community-based intervention program. In detention facilities youth are incarcerated for a period of 6–36 months; during their sentence, they can continue/complete their academic education and benefit from a structured cognitive-behavioral group program, among other kind of interventions. While an offense must be committed when a youth is between the ages of 12 and 16 years old, detained youth may be 18 years of age or older while serving sentence, because sentence lengths can last up to 3 years. In community-based intervention programs youth are assigned to an individual rehabilitation plan that can last from 6 to 24 months, which is designed and supervised by probation officers and to which they must abide while still living at home.

A random number table was used to select a sample of 250 male young offenders (125 young offenders from each group). All participants were selected during the sentencing period. Following this selection, 30 youth placed in community-based programs and 2 youth placed in juvenile detention facilities declined to participate in this study.

The final sample for this study included 217 Portuguese male young offenders. From this total sample, 122 (56.3 %) youth were placed in juvenile detention facilities (which represents 51.9 % of all young offenders placed in Portuguese juvenile detention facilities at the time of data collection) and 95 (43.7 %) youth were receiving community-based programs (which represents 21.2 % of all young offenders placed in community-based programs at the time of data collection). These 217 young offenders were then assessed with structured clinical interviews (for a description of the interviews, see the “Measures” section).

Demographic and criminal features of the total sample and groups are reported in Table 1. Groups were equivalent regarding mean age, age groups (i.e., aged 17 years or younger *vs* aged 18 years or older),^1^ socioeconomic status (SES),^2^ and repeated grade-level (i.e., number of years each participant was retained in the same school year). A significant difference between groups was observed concerning years of education; youth receiving community-based programs completed more years in school than youth placed in juvenile facilities. Groups were also compared regarding the legal category of the most severe offense for which they were sentenced, and no significant differences were observed between groups.

**Table 1:** Demographic and criminal features for the total sample and by groups

	Total sample (n = 217)		Youth placed in juvenile facilities (n = 122)		Youth receiving community-based programs (n = 95)			
	M	SD	M	SD	M	SD	t	p
Age	16.60	1.26	16.65	1.27	16.54	1.25	0.605	0.546
Years of education	6.19	1.55	5.96	1.43	6.48	1.64	−2.506	0.013
Repeated grade-level	3.02	1.37	3.11	1.33	2.91	1.41	1.066	0.288
Sentence length (in months)	18.53	6.62	19.83	7.00	16.85	5.71	–	–

	n	%	n	%	n	%	χ^2^	p
Age groups								
<18 years old	155	71.4	63	76.8	76	66.7	2.86	0.091
≥18 years old	61	28.1	18	22.0	38	33.3		
Socio-economic status								
Low	139	81.1	103	84.4	73	76.2	2.005	0.157
Medium	56	18.9	19	15.6	22	23.2		
Type of crime								
Against people	169	77.9	100	82.0	69	72.6	4.122	0.249
Against property	39	18.0	18	14.8	21	22.1		
Against life in society	8	3.7	3	2.5	5	5.3		
Drug trafficking	1	0.5	1	0.8	–	–		

Groups were not compared concerning sentence length. Crimes against life in society includes counterfeiting, forgery of documents and fire setting

### Measures

Participants were interviewed with a structured clinical interview, the MINI-KID-Mini-International Neuropsychiatric Interview for Children and Adolescents [35], which assesses Axis I mental health disorders according to DSM-IV criteria, namely: mood disorders; anxiety disorders; substance-related disorders; tic disorders; disruptive disorders and attention-deficit hyperactivity disorder; psychotic disorders; eating disorders; and adjustment disorders. The interview also has a section that allows the screening of pervasive developmental disorders. The MINI-KID can be used to diagnose mental health disorders categorically (present or absent) and dimensionally (according to the number of criteria met for each diagnosis). The MINI-KID also provides a summary sheet with a pathology profile covering the mental health disorders that the individual fulfilled criteria for, allowing the interviewer to decide which disorder should be the major focus of clinical attention (i.e., the main diagnosis). The following question is present at the end of this summary sheet profile in order to guide clinicians in this decision: “Which problem troubles him/her the most or dominates the others or came first in the natural history?”

In a previous study, inter-rater and test–retest kappas were substantial to almost perfect (0.64–1.00) for all psychopathological disorders assessed with the MINI-KID, except for dysthymia [35]. Inter-rater and test retest validity was not analyzed in this study, due to time and resources restrictions. In order to minimize, at least partially, this limitation, all interviewers attended a 3 days training in the use of the MINI-KID and had regular supervision sessions with the first author of this paper during data collection.

Participants aged 18 years or older, who met criteria for conduct disorder, were also interviewed with the antisocial personality disorder section of SCID-II-Structured Clinical Interview for DSM-IV Axis II Personality Disorders [36]. Though other personality disorders are known to be prevalent in offenders, particularly all cluster B personality disorders and paranoid personality disorder, antisocial personality disorder is the most prevalent among male offenders [37] and, as known, it must be preceded by an earlier diagnosis of conduct disorder. Taking into account these findings, and considering time and resources restrictions, the authors decided to focus on the assessment of antisocial personality disorder for those youth who already met criteria for conduct disorder.

### Procedures

The research team translated and adapted into Portuguese the MINI-KID interview [35] after obtaining permission from the authors of the original version to use the interview for research purposes. The MINI-KID was translated and adapted into Portuguese following a translation and back-translation procedure [38]. The translation was carried out by three Portuguese researchers who are fluent in Portuguese and English. They all had previous clinical practice with adolescents, which allowed them to adapt the language to this specific age group. The interview was revised by a senior Portuguese researcher to assure that questions were worded in a way that addressed the same criteria as the original version. The interview was back-translated into English by a native English speaker researcher, unrelated to this study. The back-translation was sent to the author of the original MINI-KID for revision. No significant differences were found between the back-translation and the original version, indicating that the Portuguese version of the interview had the same or very similar meaning as the original English version. The final version of the interview was then tested in a community sample of ten male youth in order to assure its suitability.

In addition to the institutional authorization from the Portuguese Ministry of Justice, all participants were informed of the goals of the study and the confidentiality and anonymity of their responses were guaranteed. Moreover, it was explained that their participation in this study would not impact their sentencing in any way. Afterwards, all participants younger than 18 years of age verbally assented to their own participation; written consent was in addition gathered from their parents/legal guardians (i.e., individuals that have legal authority to care for another person). In turn, participants older than 18 years of age provided verbal and written consent for their own participation. All young offenders were assessed individually by six of the authors of this paper, having received a three days training in the management and rating of the interviews, and regular supervision during assessment procedures.

### Data analysis

Chi square statistics were carried out using the IBM SPSS Statistics v21.0. Considering that most of the data were categorical, Chi square statistics were used in order to compare the frequencies observed in certain categories with the frequencies expected by chance in those same categories; when the expected count in each category was lower than 5, the Fisher’s exact test was considered. A significant test-value (i.e., *p* < 0.05) indicated that the distribution of frequencies across categories was potentially non-random. Standardized residuals were also analyzed as indicators of the significance of the discrepancy between observed counts and randomly/statistically expected counts; they were considered to indicate a count significantly different what would be statistically expected if >|1.96|. Finally, the z test was computed as a way to compare the proportion of the frequency of the first column that falls into a given row against the proportion of the frequency of the second column that falls into that same row [39]. Odds-ratio analyses were also carried out in order to explore how several diagnostic categories would predict belonging to one of the groups considered in the current work, using the MedCalc Easy-to-use statistical software, available at https://www.medcalc.org/calc/odds_ratio.php. Odds-ratio risk statistics were used to investigate the role of the most frequent diagnosis (i.e., conduct disorder) as an increased risk of developing additional mental health problems.

## Results

**Fig. 1:**
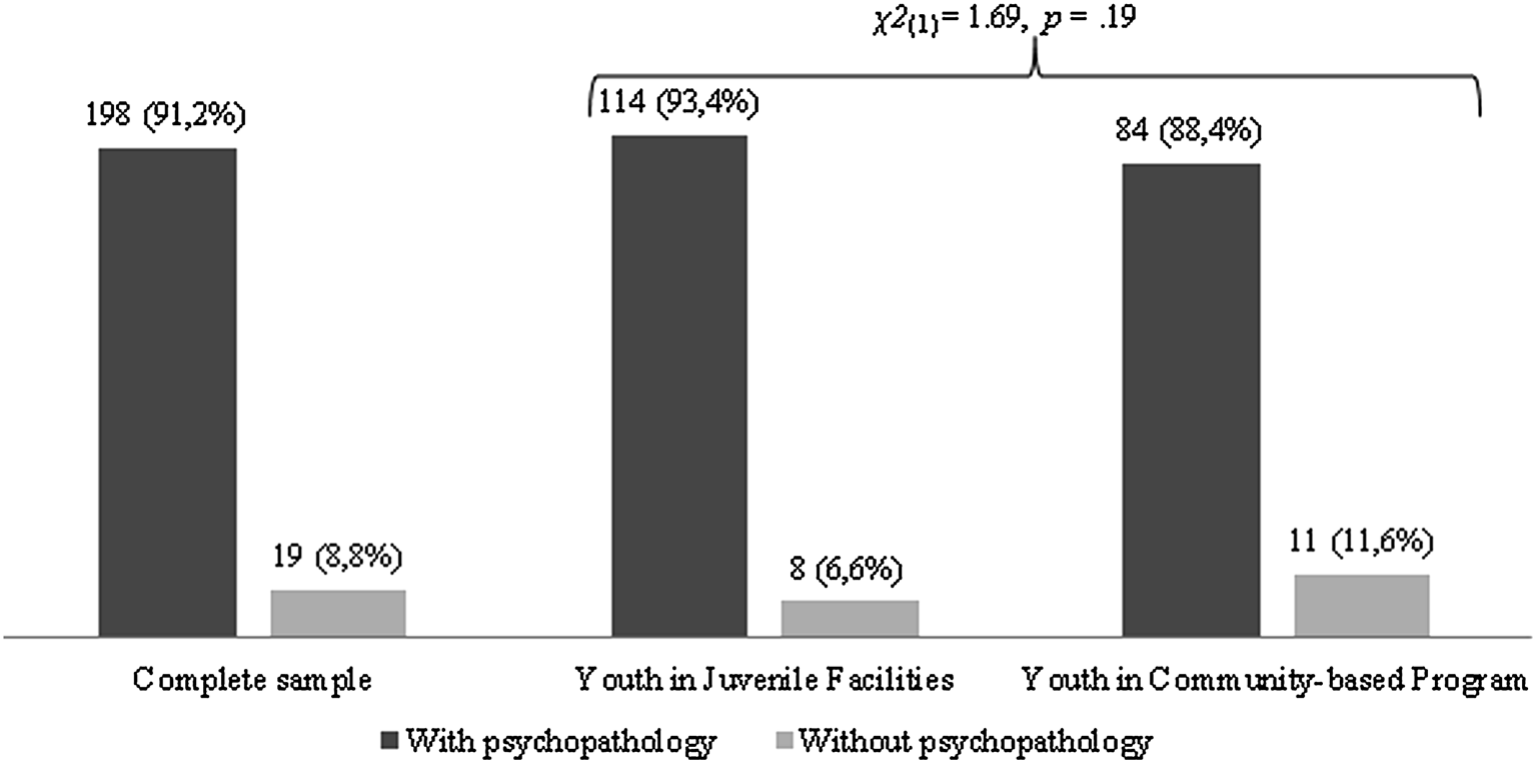
Frequency of global prevalence rate for the total sample and by groups. This figure presents the percentage of youth with and without psychopathology in the complete sample, as well as in the two groups

Figure 1 displays the global prevalence rate (i.e., participants fulfilling criteria for at least one psychiatric disorder as assessed by the MINI-KID), for the total sample and for the community and detained samples separately. Results showed a very high prevalence of mental health disorders, with 91.2 % of the total sample fulfilling criteria for, at least, one psychiatric disorder. The global prevalence rate was equally high for both groups. Also, no significant difference was found when comparing the proportion of participants with or without psychopathology in both groups (see Fig. 1).

Concerning diagnostic categories, most participants in the total sample met criteria for disruptive disorders (n = 168, 77.4 %), followed by substance-related disorders (n = 68, 31.3 %), anxiety disorders (n = 44, 20.3 %), mood disorders (n = 33, 15.2 %) and, more seldom, tic disorders (n = 4, 1.9 %). When comparing youth placed in juvenile facilities with youth receiving community-based programs (see Table 2), Chi square tests showed similar distributions according to a diagnosis of disruptive disorders and tic disorders. Regarding other diagnostic categories, the Chi square results were significant. Thus, participants from both groups were not randomly distributed for substance-related disorders, anxiety disorders, and mood disorders. Contrasting the observed versus the expected count, more participants in the community group than statistically expected presented an anxiety or a mood disorder, whereas more participants placed in juvenile facilities than statistically expected fulfilled criteria for a substance-related disorder.

**Table 2:** Frequency of the diagnostic categories by groups

	Youth in juvenile facilities			Youth in community-based programs			*χ* ^2^ statistics		Odds-ratio statistics			
	Count	Expected	Percentage	Count	Expected	Percentage	*χ* ^2^	*p*	Odds-ratio	95 % CI	*z*	*p*
Disruptive disorders	97	94.5	79.5	71	73.5	74.7	0.69	0.40	1.31	0.069; 2.48	0.83	0.41
Substance-related disorders	48	38.2	39.3	20	29.8	21.1	8.31	0.004	2.85	1.32; 4.88	2.85	0.004
Anxiety disorders	17	24.7	13.9	27	19.3	28.4	6.93	0.008	2.45	1.24; 4.84	2.59	0.009
Mood disorders	13	18.6	10.7	20	14.4	21.1	4.48	0.034	2.24	1.05; 4.79	2.08	0.037
Tic disorders	1	2.2	0.8	3	1.8	3.2	1.61	0.20	0.25	0.03; 2.48	1.18	0.24

Results are presented only for the presence of psychopathology within each diagnostic category. Disruptive disorders includes attention deficit/hyperactivity disorder, oppositional/defiant disorder, and conduct disorder. Substance-related disorders includes alcohol dependence/abuse, and substance (non-alcohol) dependence/abuse. anxiety disorders includes panic disorder, agoraphobia, separation anxiety disorder, social phobia, specific phobia, obsessive–compulsive disorder, and post-traumatic stress disorder. Tic disorders includes motor tic disorder, vocal tic disorder, and transient Tic disorder. Mood disorders includes major depression disorder, major recurrent depression disorder, and bipolar disorders

*Count* observed count, *expected* expected count, *CI* confidence interval

Considering these significant Chi square results, these diagnostic categories were further studied as predictors of belonging to one of the sample groups: the custodial group was taken as the risk group for substance-related disorders, whereas the community based group was taken as the risk group for anxiety and mood disorders (see Table 2). There was a significant co-occurrence of substance related disorders and being placed in juvenile facilities; participants fulfilling criteria for a diagnosis within this category were about three times more likely to belong to the custodial group. Alternatively, there was a significant co-occurrence of anxiety and mood disorders and belonging to the community-based group. So, participants whose main diagnosis was in either the anxiety or mood disorder categories were about two times more likely to be placed in community based-programs.

Concerning specific main diagnosis, the majority of the individuals in the total sample was diagnosed with conduct disorder (n = 128, 65 %), followed by antisocial personality disorder (n = 33, 16.8 %), oppositional defiant disorder and attention deficit hyperactivity disorder—inattentive (n = 9; 4.6 %), attention deficit hyperactivity disorder—combined and recurrent major depression (both with n = 3, 1.5 %), current bipolar disorder, post-traumatic stress disorder and attention deficit hyperactivity disorder—hyperactive (all with n = 2, 1.0 %), and, finally, current major depression, past major depression, recurrent major depression, panic disorder, agoraphobia, obsessive–compulsive disorder, and substance abuse (all with n = 1, 0.5 %).

Because the antisocial personality disorder diagnosis could only be established for participants older than 18 years of age, we further studied the main diagnosis by groups in the universe of participants who were 17 years or younger on the one hand (n = 139), and in the universe of participants who were 18 years or older on the other (n = 61); one participant taken from the community-based group did not provide information on his age and so was not included in any of these analysis (see Table 1).

When analyzing participants 17 years old or younger, the significant Fisher’s exact test result pointed to a non-random distribution of main diagnoses between youth placed in juvenile facilities and youth placed in community-based programs (see Table 3). Moreover, the z test for the proportion of frequencies in each category pointed to significantly different proportions in community versus detained youth presenting a main diagnosis of oppositional defiant disorder and conduct disorder. Specifically, the proportion of community participants presenting a main diagnosis of oppositional defiant disorder was significantly higher than the proportion of detained participants presenting such a diagnosis; inversely, the proportion of community participants presenting a main diagnosis of conduct disorder was significantly lower than the proportion of detained participants presenting such a diagnosis. No significant standardized residuals were found.

**Table 3:** Frequency of the main diagnosis by groups, for participants aged 17 years or younger

	Youth in juvenile facilities				Youth in community-based programs			
	Count	Expected	STR	Percentage	Count	Expected	STR	Percentage
Conduct disorder	68	59.6	89.5	1.1	41	49.4	65.1	−1.2
Oppositional defiant disorder	1	4.4	1.3	−1.6	7	3.6	11.1	1.8
Attention deficit hyperactivity disorder—inattentive	2	3.8	2.6	−0.9	5	3.2	7.9	1.0
Attention deficit hyperactivity disorder—combined	1	1.6	1.3	−0.5	2	1.4	3.2	0.5
Recurrent major depression	0	1.6	0.0	−1.3	3	1.4	4.8	1.4
Current bipolar disorder	0	0.5	0.0	−0.7	1	0.5	1.6	0.8
Post-traumatic stress disorder	1	0.5	1.3	0.6	0	0.5	0.0	−0.7
Attention deficit hyperactivity disorder—hyperactive	2	1.1	2.6	0.9	0	0.9	0.0	−1.0
Current major depression	0	0.5	0.0	−0.7	1	0.5	1.6	0.8
Past major depression	0	0.5	0.0	−0.7	1	0.5	1.6	0.8
Agoraphobia	0	0.5	0.0	−0.7	1	0.5	1.6	0.8
Obsessive–compulsive disorders	0	0.5	0.0	−0.7	1	0.5	1.6	0.8
Substance abuse	1	0.5	1.3	0.6	0	0.5	0.0	−0.7

Results are presented only for the presence of psychopathology within each main diagnosis. So, nine participants placed in juvenile facilities and five placed in community settings are not counted in the table because they did not fulfill criteria for any diagnoses

*Count* observed count, *expected* expected count, *STR* standardized residuals

Fisher’s exact test is significant at p = 0.001

The same analysis as applied to participants aged 18 years or older yielded a significant Fisher’s exact test (see Table 4). The z test showed a significant higher proportion of participants in the detained group as receiving a diagnosis of conduct disorder or antisocial personality disorder, in comparison with the community-based group. No significant standardized residuals were found.

**Table 4:** Frequency of the main diagnosis by groups, for participants aged 18 years or older

	Youth in juvenile facilities				Youth in community-based programs			
	Count	Expected	STR	Percentage	Count	Expected	STR	Percentage
Antisocial personality disorder	27	22.4	71.1	1.0	6	10.6	33.3	−1.4
Conduct disorder	9	12.2	23.7	−0.9	9	5.8	50.0	1.3
Oppositional defiant disorder	0	0.7	0.0	−0.8	1	0.3	5.6	1.2
Attention deficit hyperactive disorder—inattentive	1	1.4	2.6	−0.3	1	0.6	5.6	0.4
Current bipolar disorder	0	0.7	0.0	−0.8	1	0.3	5.6	1.2
Post-traumatic stress disorder	1	0.7	2.6	0.4	0	0.3	0.0	−0.6

Results are presented only for the presence of psychopathology within each main diagnosis. So, three participants placed in juvenile facilities and two placed in community settings are not counted in the table because they did not fulfill criteria for any diagnoses

*Count* observed count, *expected* expected count, *STR* standardized residuals

Fisher’s exact test is significant at p = 0.014

**Fig. 2:**
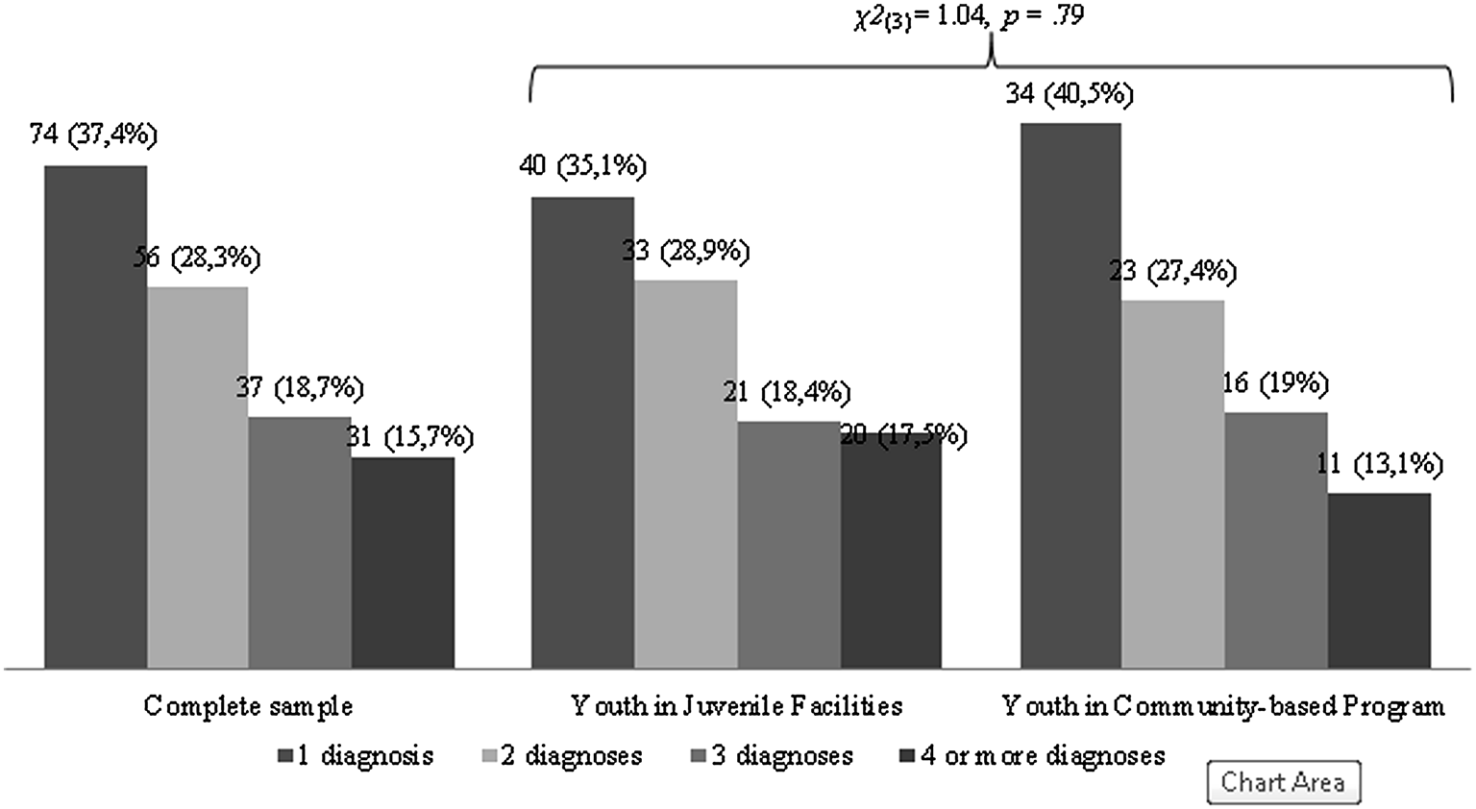
Frequency of psychiatric comorbidity for the total sample and by groups. This figure presents the percentage of youth with one, two, three and four or more diagnoses in the complete sample, as well as in the two groups

In addition to the main diagnosis, the majority of subjects fulfilled criteria for additional diagnoses (n = 124, 62.7 % for the total sample, n = 74, 64.8 % for youth placed in juvenile facilities, and n = 50, 59.5 % for youth placed in community-based programs). Both groups were similar regarding the proportion of participants presenting co-morbidities (see Fig. 2).

Due to the high prevalence of conduct disorder found in the total sample, odds ratio was computed to assess the risk of subjects with conduct disorder being diagnosed with any other axis I disorder. There was a significantly high risk of co-occurrence of conduct disorder and substance abuse: young offenders with a conduct disorder were over four times more likely to fulfill criteria for substance abuse (odds-ratio = 4.57, 95 % confidence interval for odds-ratio = 1.32; 15.93, *z* = 2.39, *p* = 0.01). The odds ratio results relating conduct disorder with all other axis I disorders were non-significant.

## Discussion

Despite available international data on the high prevalence of mental health problems in young offenders [2, 4], this study presents the first systematic assessment of mental health disorders in male young offenders intervened by the Portuguese Juvenile Justice System. Therefore, the main goal of the present study was to assess mental health problems in male young offenders, in order to identify mental health intervention needs within this population. This study adds to the few European studies on this topic, and tried to overcome some limitations of previous research. Firstly, sample size and randomized selection of participants helped to improve sample representativeness, providing for more reliable generalizations. Secondly, validated structured clinical interviews were used to establish diagnoses, making assessment procedures more standardized. Thirdly, this paper adds to the few previous studies [32] comparing prevalence rates of psychiatric disorders among a group of male young offenders placed in juvenile facilities and a group of young offenders in community-based programs.

In line with previous research [1, 3, 4, 6], results of the current study pointed out a high global prevalence rate of mental disorders among male young offenders. Nine out of ten youth fulfilled criteria for, at least, one psychiatric disorder. As expected [4–7], disruptive disorders (attention deficit/hyperactivity disorder, oppositional/defiant disorder, and conduct disorder) and antisocial personality disorder were the most frequent diagnoses in this study for both groups of young offenders: placed in juvenile facilities or receiving community-based programs.

When comparing youth placed in juvenile facilities with youth receiving community-based intervention programs, no significant difference was observed concerning the global prevalence rates. Alternatively, when considering diagnostic categories, dissimilar proportions were found by groups. On the one hand, youth placed in juvenile facilities more often received a substance related diagnosis; participants with that kind of diagnosis were, in fact, about three times more likely to be part of this custodial group. This result may be explained, at least partially, by the fact that more severe and stable forms of antisocial behavior, which in general lead youth to be placed in juvenile facilities, are more likely to be associated with a substance related diagnosis [23, 25]. On the other hand, youth placed in community-based programs were more frequently diagnosed with an anxiety or mood related disorder; participants receiving diagnosis within one of these categories were about two times more likely to be part of the community-based group. This may be due to the fact that, in Portugal, young offenders placed in community-based programs have, in general, access to fewer opportunities for having their mental health needs met, in comparison to those placed in juvenile detention facilities. In other words, the intense supervision in custody may meet, at least partially, some of the young offender’s mental health intervention needs, namely by reducing opportunities for peer and family relationship conflicts and by the use of psychotropic medication [19].

In line with previous research [4–7], and considering specific diagnoses, conduct disorder and antisocial personality disorder were the most frequent main diagnoses. We must also stress that, comparing with other studies [4, 11–14], lower prevalence rates for post-traumatic stress disorder were found. However, as D’Andrea et al. [40] argued, children exposed to trauma, as it seems to be the case of the majority of young offenders [11], often meet criteria for other psychiatric disorders rather than post-traumatic stress disorder.

Oppositional defiant disorder was more prevalent among offenders in community-based programs, while conduct disorder and antisocial personality disorder were less prevalent in offenders from that same group. Given that both groups were overall equivalent at the SES level, this result may be better explained by the fact that incarcerated youth tend to have committed more severe offences, thus fulfilling criteria for more pervasive pathology, namely antisocial personality disorder. On the other hand, it is expected that youth placed in community-based programs present a less severe type of antisocial behavior.

Similarly to what has been observed in other studies [1, 4, 5, 15, 19], a considerable high psychiatric comorbidity rate was found, either when analyzing the total sample, as when considering both groups separately, with similar proportion comorbidity rates having been found between groups. It is also worth noting that participants receiving conduct disorder as the main diagnosis were over four times more likely to have substance abuse problems. As some authors argue [15, 23], these individuals should be regarded as patients at risk of developing dual pathology in adulthood, and constitute a specific group with particular mental health intervention needs.

These results highlight several issues regarding juvenile policies. Firstly, it may be the case that the diverse services working in the prevention and early detection in community settings are not able to work together in a concerted effort, as to prevent the fact that adolescents who are signaled to the juvenile justice system show severe and pervasive psychological problems [41]. Thus, it seems of the utmost importance that the national health system, schools, and child protection services become able to identify, assess, and/or intervene effectively with at-risk children in early stages of the development. This kind of preventive policy has proven to have positive effects on preventing persistent juvenile delinquency, namely when interventions are behavioral-oriented, delivered in a family or multimodal format, and when their intensity matches the level of risk presented by the juveniles [42]. Secondly, though most young offenders either placed in juvenile detention facilities or receiving community-based programs present disruptive disorders and/or antisocial personality disorder, results highlight a considerable variability in the psychiatric symptomatology of these youth. Particularly, a considerable percentage of them also meet criteria for internalizing disorders. These results emphasize the need for an individual and rigorous mental health assessment of all young offenders intervened by the juvenile justice systems. This individual assessment procedure should be done before the court’s decision in order to inform the judge about the mental health needs of any particular young offender. Such an assessment should also help the judge to decide about the nature of the intervention provided by the juvenile justice and/or health services [17, 43]. Finally, though it is well established that recidivism risk assessment in forensic settings can provide information about the nature, intensity, and length of interventions [44, 45], the mental health paradigm can provide specific models targeting the core processes underling these youth’s dysregulation problems, which may represent possible maintenance factors of their criminal behavior and/or relevant variables concerning treatment responsiveness [46]. In other words, taking into account the young offender’s diversity of symptomatology, intervention programs should be tailored and delivered by qualified professionals. Interventions targeting these mental health needs should be a goal of any intervention effort in juvenile justice settings, especially if we take into account that individuals in this developmental phase are more responsive to treatment [27, 28].

These issues draw attention to the responsibility of decision-makers if real rehabilitation is to be achieved [1, 47, 48], namely to the scarcity of specialized facilities and services aiming to meet the needs of adolescents at the interface between mental health, protection, and criminal justice [17, 32, 49]. Regular forensic settings act mainly as controlling environments aimed primarily at security [36], not assessing nor addressing properly the mental health intervention needs of young offenders [17, 26]. As some authors emphasized [17], forensic mental health services that simultaneously assess and meet mental health and security needs of delinquent youth are essential, particularly for those with severe forms of psychopathology. The cost of ignoring the mental health needs of young offenders may be reflected, at least partially, in the high recidivism rates and the large amount of adult inmates who had previous contact with the juvenile justice system and present full-blown and pervasive clusters of mental disorders [47–50].

One clear limitation of this study is the absence of inter-rater and reliability indicators of the MINI-KID. Though we tried to minimize this limitation, with training and supervision of the interviewers, future studies should overcome this issue. It is important to add that a higher number of youth in community-based programs refused to participate in this study, when compared to youth placed in juvenile facilities. Nonetheless, it was possible to obtain representative samples of the Portuguese youth placed in either community-based programs or juvenile detention facilities that, in turn, speak well of the generality of our findings.

Another limitation was related to the exclusion criteria, namely the presence of cognitive impairment, psychotic disorders and/or pervasive developmental disorders. Research suggests that intellectual disabilities [32, 51], psychotic disorders [52], and/or pervasive developmental disorders [53] are present in young offenders, although in a low rate, and they are not always properly identified. Although youth with these specific psychiatric disorders should not be involved in regular forensic settings in the first place, research has shown that some of these youth are mistakenly/unnecessarily placed in juvenile facilities [51–53]. Therefore, early screening for those psychiatric disorders seems paramount among young offenders [51–53]. Further research should fully assess mental health needs of male and female young offenders in order to better develop specific intervention programs for those youth.

Future studies should also explore the associations between symptomatic disorders and the full range of personality disorders (not only antisocial personality disorder) in young offenders, as well as the link between mental health problems, violent behavior, and recidivism. Functional impairment along with prevalence rates of mental disorders in young offenders should also be addressed in future research, because there are only a few studies examining this issue, which is relevant to clinical practice and policy decisions [15].

## Conclusions

Overall, our findings pointed out the need to take into account specific mental health intervention needs in male young offenders when deciding and planning any forensic intervention. It seems of the utmost importance to promote early detection and more effective intervention at a preventive level. Additionally, early screening, followed by a standardized assessment protocol to evaluate mental health problems of young offenders, seems a major requirement. It seems paramount to design psychotherapeutic interventions that tack the mental health intervention needs of young offenders. The development and delivery of intervention programs should be thoroughly assessed, so research can inform the ongoing clinical practice and vice versa. Finally, it appears important to establish a link with community-based mental health services at the end of the intervention by the juvenile justice services. All of these implications are relevant for both young offenders placed in juvenile facilities and youth receiving community-based programs, in looking for the improvement of current practices of the national health and justice systems.

## Abbreviations

MINI-KID: Mini-International Neuropsychiatric Interview for Children and Adolescents
SCID-II: Structured Clinical Interview for DSM-IV Axis II Personality Disorders
SES: socioeconomic status

## Footnotes

1 The split of the sample into two age groups is relevant taking into account the differential use of assessment instruments (cf. see “Measures” section).

2 SES was measured by parents’ profession, considering the Portuguese professions classification [34]. Examples of professions in the high SES group are judges, higher education professors, or MDs; in the medium SES group are nurses, psychologists, or school teachers; and in the low SES group are farmers, cleaning staff, or undifferentiated workers.
